# Conformational Dynamics of Herpesviral NEC Proteins in Different Oligomerization States

**DOI:** 10.3390/ijms19102908

**Published:** 2018-09-25

**Authors:** Benedikt Diewald, Eileen Socher, Christian A. Söldner, Heinrich Sticht

**Affiliations:** Division of Bioinformatics, Institute of Biochemistry, Friedrich-Alexander-Universität Erlangen-Nürnberg (FAU), Fahrstraße 17, 91054 Erlangen, Germany; benedikt.diewald@fau.de (B.D.); eileen.socher@fau.de (E.S.); christian.soeldner@fau.de (C.A.S.)

**Keywords:** molecular dynamics, HCMV, HSV-1, PRV, nuclear egress complex, α-herpesvirus, β-herpesvirus

## Abstract

All herpesviruses use a heterodimeric nuclear egress complex (NEC) to transport capsids out of host cell nuclei. Despite their overall similar structure, NECs may differ significantly in sequence between different viruses. Up to now, structural information is limited to isolated NEC heterodimers and to large hexagonal lattices made up of hexagonal ring-like structures (“Hexagons”). The present study aimed to expand the existing structural knowledge with information on the dynamics of NECs from different viruses and in different oligomerization states. For this task, comparative molecular dynamics simulations were performed of the free NEC heterodimers from three different viruses (HCMV (human cytomegalovirus), HSV-1 (herpes simplex virus 1), and PRV (pseudorabies virus)). In addition, higher oligomerization states comprising two or six NEC heterodimers were characterized for HCMV and HSV-1. The study revealed that the isolated NEC heterodimers from α- (HSV-1, PRV) and β-herpesviruses (HCMV) differ significantly in their dynamics, which can be attributed to a poorly conserved interface region between the NEC subdomains. These differences become smaller for higher oligomerization states, and both HCMV and HSV-1 individual Hexagons exhibit a common region of enhanced dynamics, which might be of functional relevance for the formation of curved vesicle structures or the recognition of hexameric capsid proteins.

## 1. Introduction

The ability of a virus to accumulate new virus particles and efficiently release them from the host cell is crucial for the infection of new cells and its persistence in an organism. An intimate understanding of viral replication and proliferation is essential for developing novel treatments. Many infectious virus particles are enveloped in the host cell membrane to form so-called enveloped viruses. The typical process that adds the lipid envelope to the virus is called budding, and can happen at the plasma membrane or at any intracellular membrane, such as endoplasmic reticulum [1] (ER), Golgi [2,3], or inner nuclear membrane (INM) [4].

Herpesviruses are enveloped DNA viruses, and present major human pathogens with a worldwide distribution and high seroprevalence in many populations [5,6]. Symptoms can range from skin lesions to more serious ailments, such as encephalitis and keratitis. Herpesviral infections are also a leading cause of hearing loss in infants [7]. Herpesviruses have the peculiar trait of budding twice, first at the INM after the capsid has been assembled in the nucleus, and second, at cytoplasmic membranes derived from the *trans*-Golgi network [8,9,10]. Additionally, budding at the nuclear membrane makes herpesviruses unique, since it has not yet been observed in other viruses. Due to their large genomes, herpesviral capsids are too big to exit the nucleus via the pores in the nuclear envelope. Therefore, they have to bud at the INM, forming enveloped viral particles in the perinuclear space that subsequently fuse with the outer nuclear membrane (ONM) to release naked capsids into the cytosol. This way, the virus can escape the nucleus to be surrounded by tegument proteins and acquire its final envelope in the second budding step [11].

**Figure 1:**
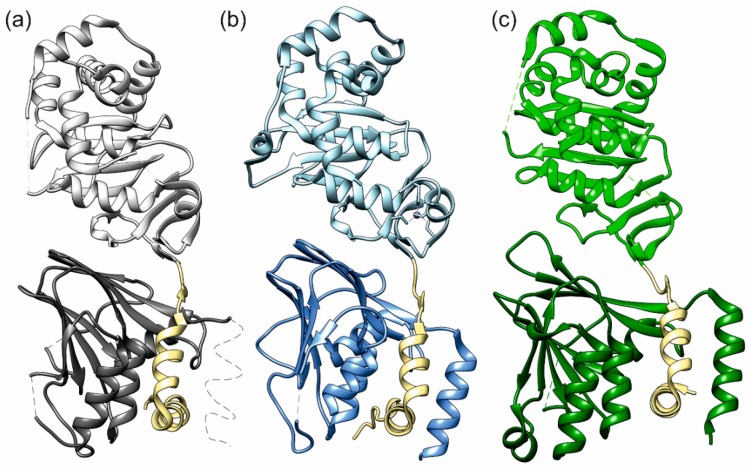
NECs (nuclear egress complexes) of the three herpesviruses (**a**) HSV-1 (herpes simplex virus 1), (**b**) PRV (pseudorabies virus), and (**c**) HCMV (human cytomegalovirus). The membrane proximal proteins (pUL50, pUL34) are in a darker shade than the lightly coloured membrane distal proteins (pUL53, pUL31). The hook regions are coloured khaki. Thin dotted lines indicate protein segments not resolved in the crystal structures.

The key component of the molecular machine that facilitates budding at the INM is called nuclear egress complex (NEC). The NEC is a heterodimeric complex of two viral proteins located on the inside of the nuclear envelope. The proteins of the NEC are designated differently among various herpesviruses. In the α-herpesviruses, herpes simplex virus-1 (HSV-1) and pseudorabies virus (PRV), the membrane-anchored NEC protein is called pUL34, and in the β-herpesvirus, human cytomegalovirus (HCMV), it is called pUL50. It binds a soluble protein named pUL31 in HSV-1 and PRV, or pUL53 in HCMV [12]. The NEC is capable of recruiting viral and cellular kinases to soften or dissolve the nuclear lamina [13,14]. However, it has been shown that, in some cases, the NEC can self-sufficiently bud through the INM and form primary envelopes in the perinuclear space [15,16]. In return, the absence of either of the NEC proteins impairs viral replication and prevents most capsids from exiting the nucleus [17,18]. Recently determined crystal structures display very similar geometries among NECs, despite their rather low sequence similarity [19,20,21,22] (Figure 1).

**Figure 2:**
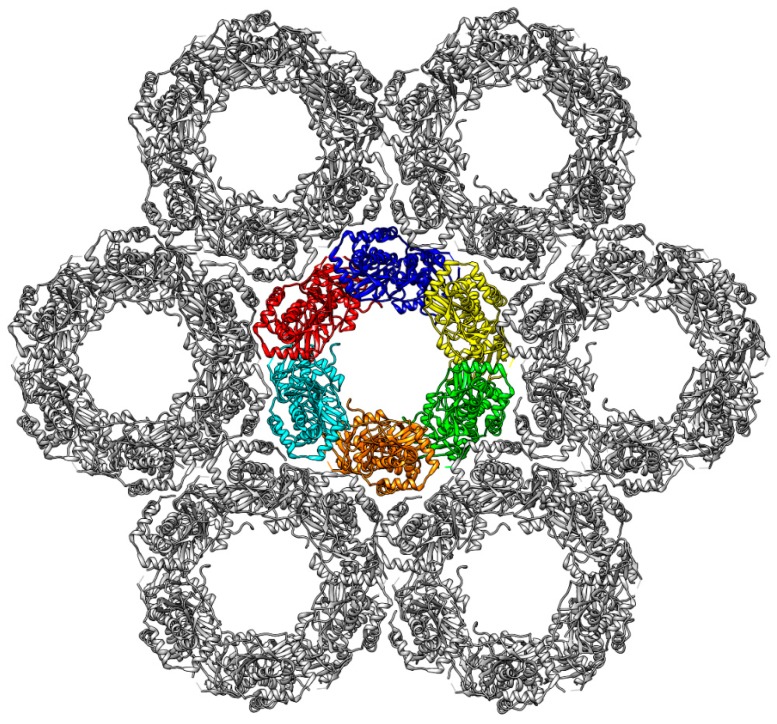
Structure of the PRV hexagonal lattice derived from a cryo-EM (cryo-electron microscopy) study [23]. In the central Hexagon, each of the six heterodimers is coloured individually (blue, yellow, green, orange, cyan, and red). Each Hexagon is surrounded by six additional Hexagons (grey), thereby forming large lattice-like structures.

The most prominent feature all those NECs have in common is the hook–groove interaction, which is formed by a hook of pUL53 (pUL31) that binds into a groove in pUL50 (pUL34) (Figure 1). The NECs of HSV-1 and HCMV crystallize in a peculiar pattern, forming large lattices made up of hexagonal ring-like structures (“Hexagons”) consisting of six NEC heterodimers each. A similar pattern has been observed for the PRV NEC when being bound to a lipid membrane [23] (Figure 2). To date, structural information is limited to the isolated NEC heterodimers and the lattices detected by X-ray crystallography and cryo-electron microscopy (cryo-EM), but no other oligomerization states have been characterized. 

This paucity of structural information renders it difficult to assess the physiological relevance of the individual Hexagons as functional units, which is a matter of discussion in the literature [24,25].

To investigate the structure and dynamics of the NEC in different oligomerization states, we have performed comparative molecular dynamics (MD) simulations of the free NEC heterodimers from three different viruses (HCMV, HSV-1, and PRV). In addition, higher oligomerization states comprising two or six NEC heterodimers were characterized for HCMV and HSV-1. The study revealed that the isolated NEC heterodimers from α- and β-herpesviruses differ significantly in their dynamics, and that these differences become smaller for higher oligomerization states. Nevertheless, even the Hexagons still exhibit significant dynamics, which might be of functional relevance for the formation of curved structures or the recognition of interaction partners. This knowledge about the conformational stability of different oligomerization states might be helpful in future to understand the pathways of NEC oligomer formation and to interfere with this process.

## 2. Results and Discussion

### 2.1. Interface Analysis of the NECs (Nuclear Egress Complexes)

**Figure 3:**
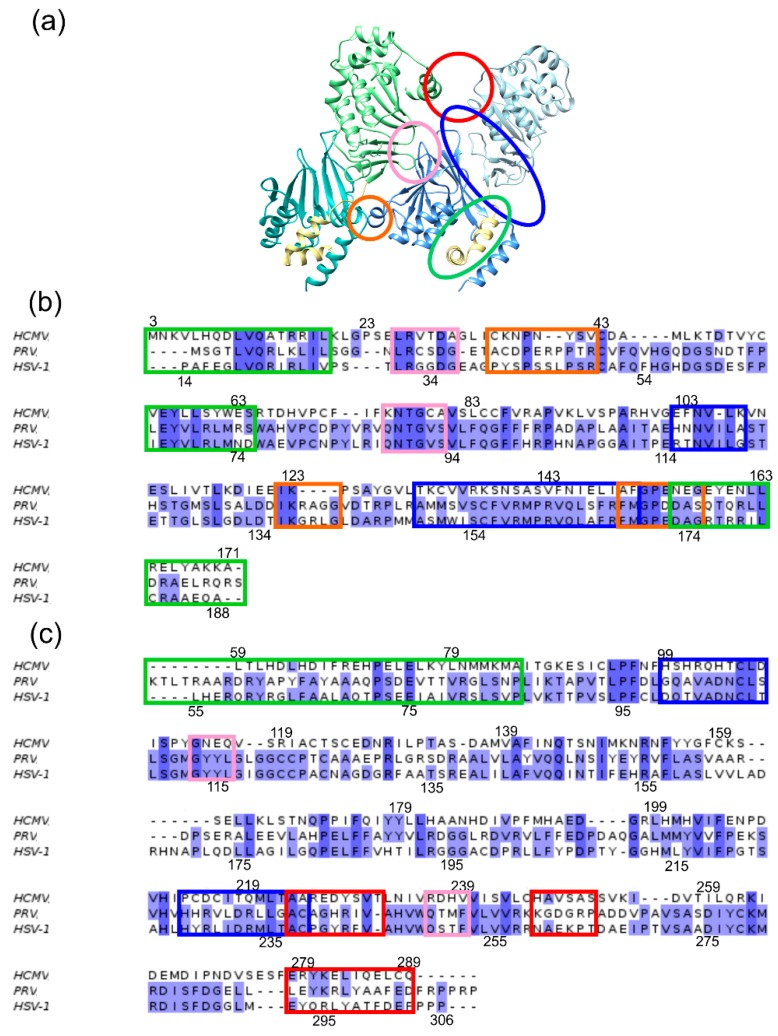
(**a**) Representation of a section from the Hexagon showing two adjacent HCMV NEC heterodimers. The proteins are coloured according to the scheme established in Figure 1. The interfaces are marked by coloured ellipses: PIF (green), SIF (blue), OIF I (red), OIF II (pink), OIF III (orange). (**b**) Structure-based sequence alignment of pUL50 (pUL34). The sequence regions of the interfaces are framed according to the established colour scheme. The letters are highlighted according to the percentage of sequence identity. (**c**) Structure-based sequence alignment of pUL53 (pUL31). Colour coding as in panel (**b**). Sequence positions for the HCMV and HSV-1 NEC are provided above and below the sequence alignments.

To allow for a comprehensive investigation of NEC oligomer stability, the interfaces were first classified according to their location and the binding partners involved (Figure 3). Within each NEC heterodimer, there exists the hook-into-groove interaction (henceforth termed “primary interface” (PIF)), as well as a structurally less well-conserved interaction site between the globular domains of pUL50 (pUL34) and pUL53 (pUL31) (henceforth termed “secondary interface” (SIF)). In addition, there are three major sites of interactions between adjacent heterodimers within the Hexagons (henceforth termed “oligomer interface”, (OIF)). OIF I connects two neighbouring pUL53 (pUL31) moieties, OIF II connects pUL53 (pUL31) of one dimer to pUL50 (pUL34) of a neighbouring dimer, and OIF III connects two neighbouring pUL50 (pUL34) units (Figure 3).

### 2.2. The HCMV NEC Heterodimer Is Considerably More Flexible Than Its Counterparts in PRV and HSV-1

**Figure 4:**
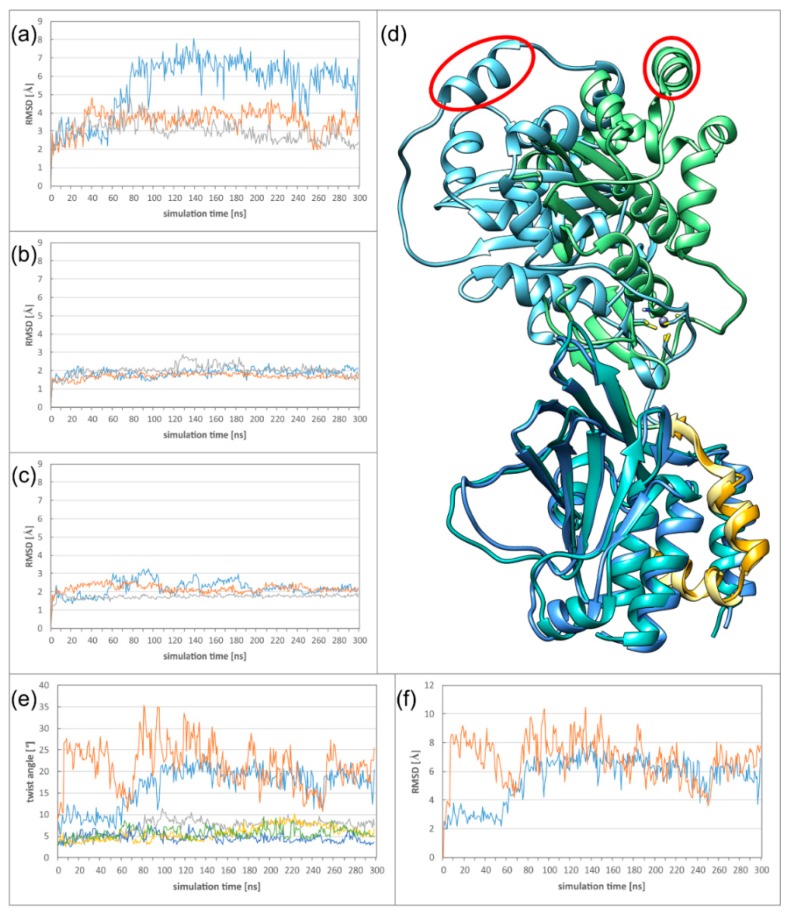
Dynamics of the NEC heterodimers (**a**) RMSD (root-mean-square deviation) plot of the complete NECs of HCMV (blue), HSV-1 (orange), and PRV (grey). (**b**) RMSD plot of the pUL53 (pUL31) subdomain of HCMV (blue), HSV-1 (orange), and PRV (grey). (**c**) RMSD plot of the pUL50 (pUL34) subdomain of HCMV (blue), HSV-1 (orange) and PRV (grey). (**d**) Overlay of the HCMV NEC starting structure and the twisted structure after 120 ns simulation time. The proteins are coloured according to the scheme established in Figure 1. One of the pUL53 helices is marked by a red ellipse to illustrate the extent of the twist. (**e**) Plot illustrating the twist in the two simulation runs of HCMV (blue and orange), HSV-1 (grey and yellow), and PRV (green and dark blue). (**f**) RMSD plot of the complete HCMV NEC in two runs.

α- and β-herpesviral NECs differ considerably in their sequences, but still have highly similar structures. To assess whether their dynamics are also similar, molecular dynamics simulations of free heterodimers in solution have been performed for the NECs of HCMV, HSV-1, and PRV. As an initial indicator for protein flexibility, the RMSD (root-mean-square deviation) values have been measured and plotted against the simulation time. The HCMV NEC displayed a much higher RMSD from the starting structure than HSV-1 and PRV in both simulation runs (Figure 4a, Figure S1 for run 2). To identify the structural origin for this difference, the complexes where split into two “subdomains”: the pUL53 subdomain containing the core part of pUL53 (pUL31), and the pUL50 subdomain containing pUL50 (pUL34) plus the hook of pUL53 (pUL31). When the subdomains are analysed separately, the RMSD of HCMV is in the same range as observed for HSV-1 and PRV (Figure 4b,c, Figures S2 and S3 for run 2), indicating that a larger movement between the HCMV subdomains is responsible for the high overall HCMV RMSD. The type of movement can be illustrated by overlaying the starting structure with the structure at 120 ns, while only fitting on the pUL50 subdomain (Figure 4d, Supplementary Materials Movie M1). Even though there are no large conformational changes in pUL50 and the hook, the pUL53 subdomain is twisted away from its starting position, which can be seen best at the highlighted helix.

The twist can be quantified by calculating the average angle of every Cα atom around a predefined axis between a reference frame and any frame of the simulation. For this purpose, an axis was defined between one Cα with low RMSF (root-mean-square fluctuation) of each subdomain (see Methods for details). When plotting the average twist angle versus the simulation time, it becomes apparent that twisting >10 degrees is exclusively observed for the HCMV NEC (Figure 4e). The highly similar time course of twist and RMSD classifies twisting as the main mode of movement in the complex (Figure 4e,f). For the α-herpesviruses, twist angles are in the range of 5 to 10 degrees (Figure 4e), which is in line with the lower RMSDs observed.

### 2.3. The Secondary Interface Is a Critical Determinant of Subdomain Orientation

The differences in NEC twisting do not arise from the primary hook–groove interaction, which is very rigid in all three herpesviral NECs throughout all simulations, but rather from the secondary interface (SIF) between the globular subdomains. In the α-herpesviruses, the SIFs are more tightly packed and exhibit a larger number of salt bridges.

**Figure 5:**
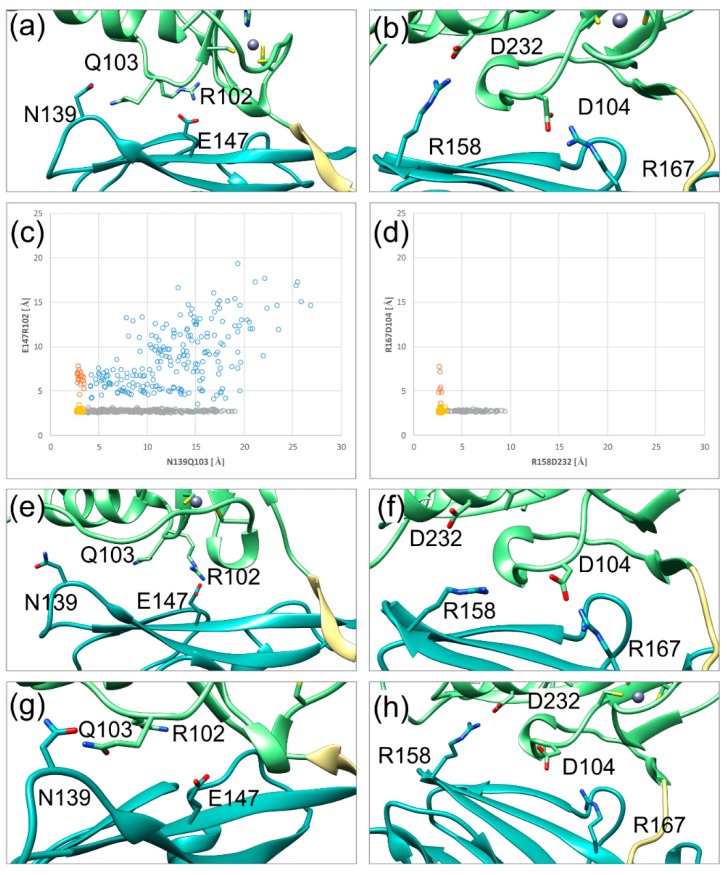

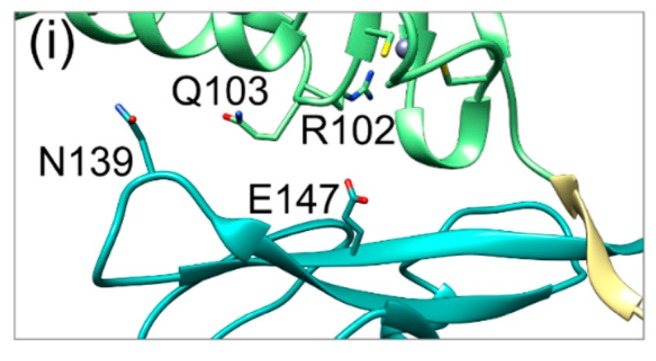
Fluctuations of the SIF (secondary interface) over the simulation time in HCMV (left) and HSV-1 (right) NECs. The pUL50(pUL34) subdomain, pUL53(pUL31) subdomain, and hook region are coloured in cyan, green, and khaki, respectively, in agreement with the scheme established in Figure 1. (**a**) The HCMV NEC SIF with two stable polar interactions. (**b**) HSV-1 NEC SIF with two stable polar interactions. (**c**) Distribution of the distances of both HCMV NEC SIF polar interactions against each other; the yellow dots represent two stable interactions, the grey and orange dots correspond to one stable interaction each, the blue dots indicate no stable interaction. (**d**) Distribution of the distances of both HSV-1 NEC SIF polar interactions against each other; colour coding as in (**c**). (**e**) HCMV NEC SIF conformation, in which the N139Q103 interaction distal to the hook is lost. (**f**) HSV-1 NEC SIF conformation, in which the D232R158 interaction distal to the hook is lost. (**g**) HCMV NEC SIF conformation, in which the R102E147 interaction proximal to the hook is lost. (**h**) HSV-1 NEC conformation, in which the D104R167 interaction proximal to the hook is lost. (**i**) HCMV NEC SIF, in which both polar interactions are lost. This situation is only observed for HCMV, but not for HSV-1.

In HCMV, there is only a single salt bridge between E147 (pUL50) and R102 (pUL53), which is located proximal to the hook (Figure 5a). This interaction has a structural analogue in HSV-1 between R167 (pUL34) and D104 (pUL31) (Figure 5b). One significant difference in the secondary interface is the second polar interaction distal from the hook: HSV-1 has a salt bridge between R158 (pUL34) and D232 (pUL31), while HCMV only has a hydrogen bond between N139 (pUL50) and Q103 (pUL53). Since the PRV NEC heterodimer displays the same structural and dynamical features as the HSV-1 NEC, details concerning the SIF will not be shown here. An analysis of the stability of the polar interactions over the simulation time revealed significant differences. The N139Q103 hydrogen bond and the E147R102 salt bridge of HCMV are only formed over 11% and 61% of the simulation time respectively, whereas the salt bridges of the HSV-1 secondary interface both persist for more than 80% of the simulation time (84% for R158D232 and 99% for R167D104). In both NECs, the polar interaction located proximal to the hook is more stable than the distal one.

Distance plots of both polar interactions against each other illustrate the difference in stability and the co-occurrence of both interactions (Figure 5c,d). In HSV-1, both salt bridges are simultaneously present for most of the simulation time (yellow dots in Figure 5d). When the salt bridges are lost, the sidechain distances can reach up to ~9 Å (orange and grey dots in Figure 5d). Notably, at least one of the salt bridges remains intact during the simulation time, thus offering a structural explanation for the rather high rigidity of domain orientation.

By contrast, the plot for the HCMV NEC shows a fourth region, in which both interactions are lost (blue dots in Figure 5c). In addition, the distance between N139 and Q103 can increase up to very high values of almost 20 Å, even when the proximal E147R102 salt bridge is present (grey dots in Figure 5c). These observations are in line with the poorer interface packing and larger conformational freedom for the subdomain orientation in HCMV compared to HSV-1 (Figure 5c). Representative interface regions resulting from the loss of one or two polar interactions are depicted in Figure 5e–i.

The results presented, so far, suggest subfamily-specific dynamics for the SIF of heterodimeric NECs. While the SIF of α-herpesviral NECs appears to be rather rigid, the SIF of the HCMV NEC is highly flexible by itself, and lacks the strong polar interactions of HSV-1 and PRV. In contrast to the PIF, which possesses a multitude of essential interactions in all NECs, essential residues in the secondary interface have only been reported for the α-herpesviral NECs. In particular, mutations of the two salt bridges have been shown to abolish pUL34-pUL31 binding altogether [26,27].

### 2.4. HCMV NEC Secondary Interface Can Be Stabilized by Introducing an Extra Salt bridge

**Figure 6:**
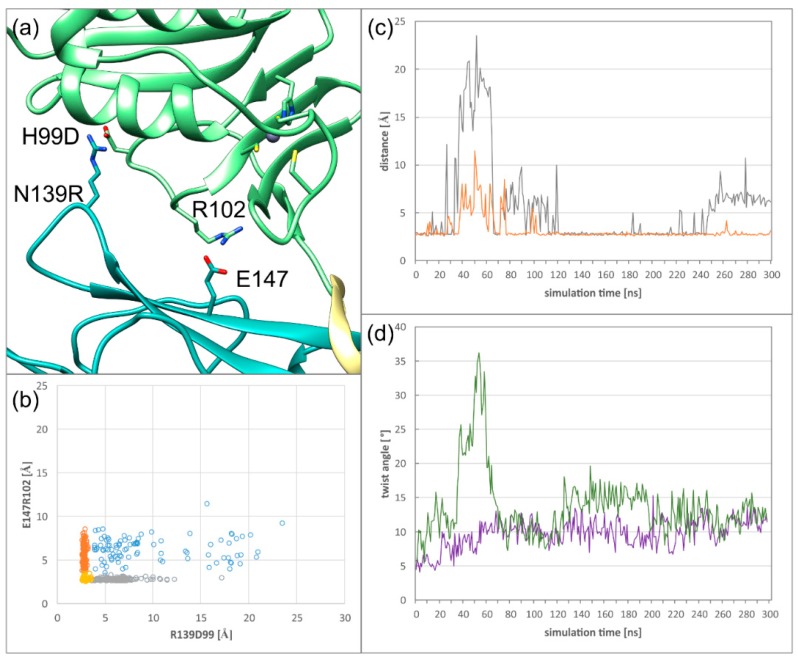
(**a**) Close view of the secondary interface of the mutant HCMV NEC, highlighting the salt bridges R102E147 and R139D99. (**b**) Distribution of the stability of the salt bridge E147R102, which is already present in the wildtype structure, against the additional salt bridge R139D99 created by mutation. (**c**) Plot of the distance of the salt bridges R139D99 (grey) and E147R102 (orange) versus simulation time (run 2). (**d**) Twist angle of both simulations run of the mutant HCMV NEC (run 1, purple; run 2, green).

To test the hypothesis, that the lack of a salt bridge distal to the hook in the secondary interface is responsible for the twist between subdomains in the HCMV NEC, a salt bridge was introduced at a suitable site. By mutating N139 of pUL50 to arginine and H99 of pUL53 to aspartate (Figure 6a), a significantly more stable interaction (61% persistence) can be formed. Interestingly, the stability of the second (E147R102) salt bridge is almost unaffected (Figure 6b). However, a simultaneous loss of both interactions is a rare event (Figure 6b,c), which corresponds to a transient conformational instability observed in run 2, from 30 to 80 ns (Figure 6c, orange line), which is characterized by a large twist angle (Figure 6d). Thus, there is a strong correlation between the simultaneous loss of both salt bridges and the occurrence of large twist angles (Figure 6c,d).

The newly introduced salt bridge is strong enough to reduce the average twist between subdomains compared to the wildtype NEC (Figure 4e). Even though the introduction of R139D99 reduces the twist between the subdomains, the absolute angle value is still higher than in HSV-1. This suggests that additional interface properties of HSV-1 further reduce the dynamics between the subdomains.

### 2.5. Dyamics of Higher NEC Oligomers

As representatives of higher oligomers, dimers of the heterodimeric NEC (termed “DimerDimer”), and hexagonal ring-structures comprising six NEC heterodimers (termed “Hexagon”), were investigated.

**Figure 7:**
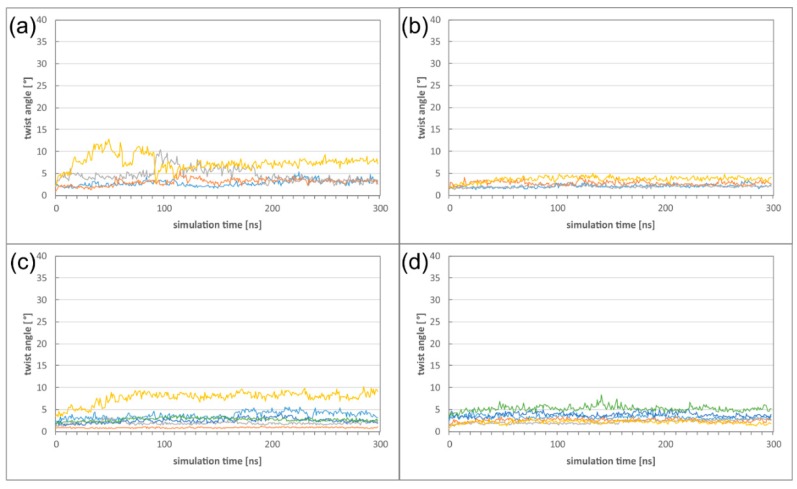
(**a**) Twist angle of the HCMV DimerDimer simulations; the two dimers of simulation run 1 are displayed in blue and orange, the two dimers of simulation run 2 are displayed in grey and yellow. (**b**) Twist angle of the HSV-1 DimerDimer simulations; the two dimers of simulation run 1 are displayed in blue and orange, the two dimers of simulation run 2 are displayed in grey and yellow. (**c**) Twist angle of the HCMV Hexagon simulations (run 1); the different colours mark the individual heterodimers. (**d**) Twist angle of the HSV-1 Hexagon simulations (run 1); the different colours mark the individual heterodimers.

First, we investigated, in which way the formation of these higher oligomers affects the heterodimer dynamics. Comparison of the twist angles to those of the isolated heterodimers (Figure 4e) reveals the DimerDimer formation results in a reduction of the twist angles both for HCMV and HSV-1, and that the effect is much more pronounced for HCMV (Figure 7a,b). Formation of Hexagons does not result in a further decrease of flexibility for both viruses, indicating that a certain degree of dynamics is even retained in these higher oligomers (Figure 7c,d; Figures S4 and S5). It is also noticeable that the large differences in twisting that have been observed between HCMV and HSV-1 NECs on the heterodimer level (Figure 4) have almost completely vanished on the level of the Hexagons.

To complement the analysis of higher oligomers, we also characterized the oligomer interfaces (OIFs), which emerge between adjacent NEC heterodimers, in more detail. Our analysis of the static crystal structures has already shown that there are three interface patches (termed OIF I to OIF III) that connect two dimers to each other, separated by regions without contacts (Figure 3).

To investigate the dynamics of these patches, each OIF was represented by one key interaction (Table 1). Comparing the OIF stability between the DimerDimer and the Hexagon reveals that the formation of rings has only a rather small effect on the stability of the interactions (Table 1). This is an unexpected finding, because one would expect that the closure of a ring confers significant rigidity to the interfaces.

When comparing the HCMV and HSV-1 oligomers, the interface patch with the lowest stability is generally OIF I (pUL53 or pUL31 homomeric interaction). Quantitatively, OIF I is even less stable in HCMV compared to HSV-1 (Table 1). One explanation for this difference might be the lower conformational stability of the secondary interface in HCMV, which favours a distortion of the globular pUL53 subdomain. Additionally, the loop containing K262 (OIF I) is longer in HSV-1 compared to HCMV, which might facilitate salt bridge formation with the adjacent pUL31 subunit.

**Figure 8:**
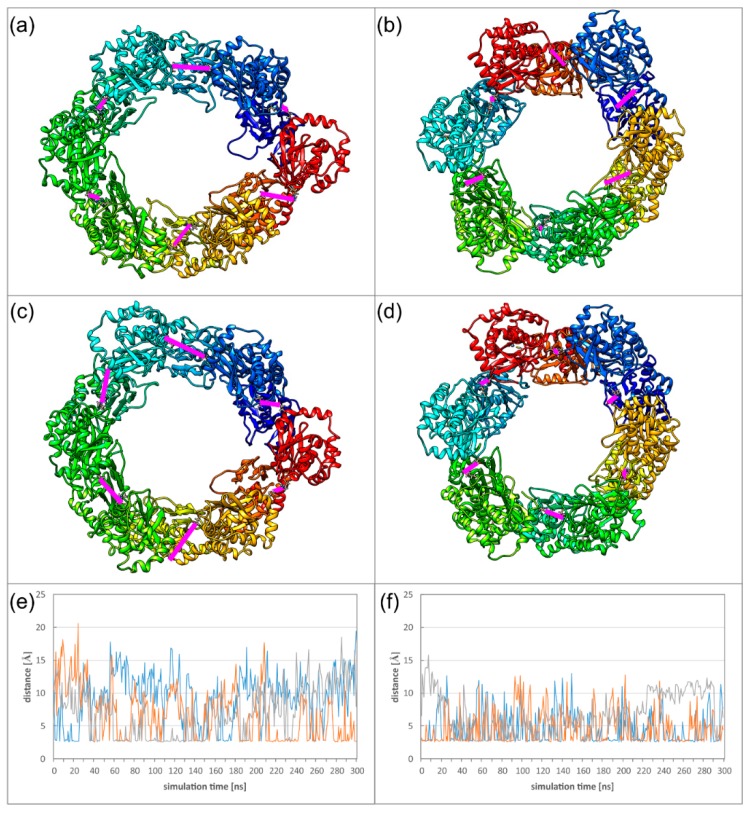
(**a**–**d**) Two alternative conformations of the HCMV (**a**,**c**) and HSV-1 (**b**,**d**) Hexagons observed during the MD (molecular dynamics) simulations. Each chain is coloured individually. Inter-subunit distances between E282R225 (HCMV) or E302K262 (HSV-1) of OIF I are highlighted with pink bars. For three of these six OIF I interactions, the time course is displayed in panels (**e**,**f**): HCMV (**e**), and HSV-1 (**f**).

A closer inspection of the salt bridge in OIF I reveals that the dynamics of the Hexagon hampers occurrence of all six salt bridges at the same time. In the HCMV Hexagon, there are mostly between one and three salt bridges present simultaneously; in the HSV-1 Hexagon it is usually between two and four. In the diagram (Figure 8), the distances of three selected inter-subunit salt bridges are plotted for each virus to illustrate this behaviour. Another difference between HCMV and HSV-1 is the exchange frequency of these interactions, which is slower for HCMV, since the movements of the pUL53 subdomain are larger than the movements of the pUL31 subdomain. The structures (Figure 8) depict two conformations for each NEC Hexagon with different distributions of salt bridges.

The relatively low stability of the oligomer interfaces is also reflected in the overall dynamics of the Hexagons. To monitor the dynamics of the Hexagons, we measured the distances between those subunits located on opposite sides of the Hexagon, thus, roughly representing the diameter of the Hexagon. In case of a rigid ring-like geometry, these distances are expected to be of similar magnitude and to exhibit only small variations over time. We defined two different types of distances to address (1) the overall deformation of the Hexagon, and (2) the motions of the pUL53 (pUL31) subdomains connected by the particularly weak OIF I.

**Figure 9:**
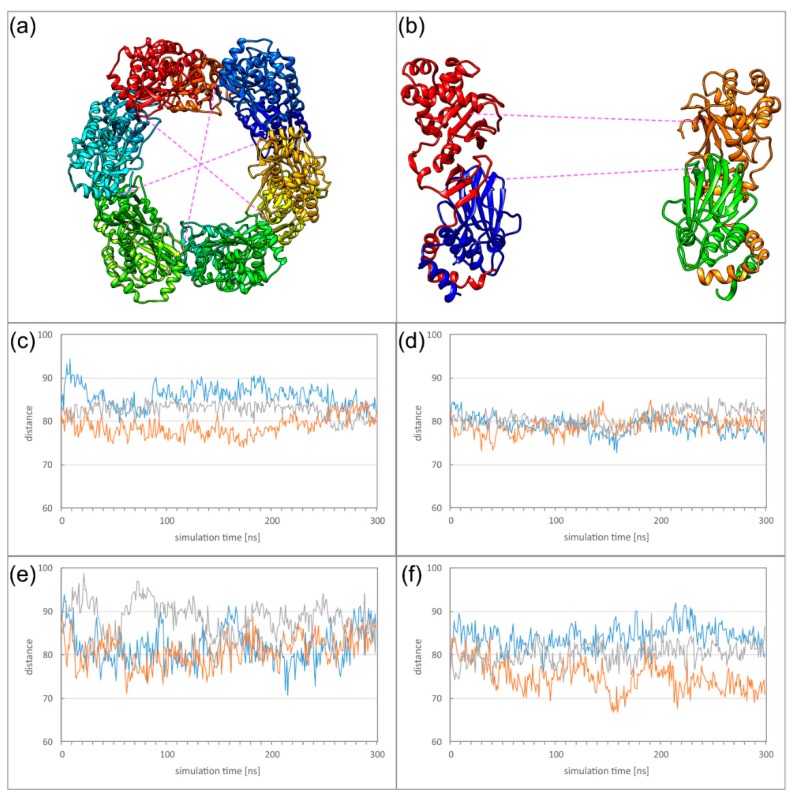
(**a**) Schematic presentation showing between which subunits ring deformation was measured. (**b**) Side view on two subunits located on opposite sides of the Hexagon. The distances analysed for global deformation (lower dotted line) and pUL53 (pUL31) flexibility (upper dotted line) are indicated. (**c**–**f**) The diagrams display the overall deformation of the ring for HCMV (**c**) and HSV-1, (**d**) as well as the flexibility of the pUL53 (pUL31) subdomains for HCMV (**e**) and HSV-1 (**f**).

For the overall deformation, distances were measured between Cα atoms of V134 for HCMV or T90 for HSV-1. These residues are located in the globular pUL50 (pUL34) domain that exhibits a high conformational stability in the isolated subunits (Figure 9a,b). In HSV-1, the three measured diameters are of roughly the same distance and oscillate by less than 5 Å (Figure 9d). The HCMV Hexagon is more dynamic and displays fluctuations of up to 10 Å for the diameter over simulation time (Figure 9c).

To assess the dynamics of the pUL53 (pUL31) subdomains, distances were measured between the Cα atoms of M201 for HCMV or L215 for HSV-1 (Figure 9b). For the dynamics of these subdomains, HCMV and HSV-1 display a rather similar behaviour (Figure 9e,f). The rather large fluctuations are in line with the low stability of the salt bridges in OIF I. The dynamics of the HCMV and HSV-1 Hexagons can also be seen from the movies provided as supplementary material (Supplementary Materials Movies M2 and M3).

## 3. Methods

### 3.1. Processing of X-ray Structures

In the present study, the NECs of three different herpesviruses (Figure 1) were studied by MD simulations: the crystal structures of the pUL34/31 complexes of HSV-1 and PRV (PDB (protein data bank) accession codes 4ZXS and 4Z3U) [20], and the pUL50/53 complex of HCMV (PDB accession code 5D5N) [19]. The PRV pUL34/31 crystal structure had been determined using a selenomethionine derivative, therefore, all selenomethionine residues where replaced with regular methionines prior to the MD simulations. Furthermore, the C-terminal stretch of HSV-1 pUL34 (residues 175–188) had not been resolved in the crystal, and was modelled in a helical conformation according to its pendant in PRV (residues 161–174) after confirming the helix propensity of this stretch by secondary structure prediction [28]. Missing loop residues in all three complexes (HSV-1: residues 38, 106 of pUL34, residues 129–133, 263–268 of pUL31; PRV: residues 1–3, 23–25,175–176 of pUL34, residues 101–102, 229–231 of pUL31; HCMV: residues 91–97 of pUL50, residues 128–130, 249–254 of pUL53) were also modelled. For these tasks, the program MODELLER [29] and the ModLoop web service [30,31] were used. N- and C-termini were capped with acetyl and *N*-methylamide respectively, unless they represented the physiological terminus of the protein. Larger oligomers were reconstructed using the symmetry information from the respective crystal structures.

### 3.2. MD Setup

The program AMBER 14 was used for all molecular dynamics simulations [32], in conjunction with the ff14SB force field [33] and the particle mesh Ewald method for the electrostatic interactions [34,35]. To neutralize charges, sodium or chloride counter ions were added. A truncated octahedral water box with TIP3P water model [36] was generated with a distance of at least 10 Å, and in the case of the HCMV NEC simulations, at least 25 Å between any atom of the complex and the fringe of the periodic boundary box. All bonds involving hydrogen atoms were constrained using the SHAKE algorithm [37]. Minimization was performed in three subsequent steps with the SANDER module of AMBER, as described previously [38]. After that, the systems were equilibrated to 310 K using a two-step protocol: In the first 25 ps step, the complex was kept fixed with weak 10 kcal·mol^−1^·Å^−2^ restraints, and heated from 0 K to 300 K using a Langevin [39,40] thermostat. Initial velocities were generated from a Boltzmann distribution using a random seed. Periodic boundaries were used with a constant volume and non-bonded interactions had a 10 Å cutoff. The second step of the equilibration took 25 ps to heat the systems from 300 K to 310 K. In this step, the restraints were lifted, and constant pressure periodic boundaries with an average pressure of 1 atm were in use. After these initial stages, the production stage was executed using constant pressure periodic boundaries with a Berendsen [41] thermostat at 310 K. Coordinates were saved every 4 ps in a trajectory over 300 ns for each system. In the cases of the larger oligomers, coordinates were saved every 10 ps.

### 3.3. Twist Angle Calculation

The average twist angle of all atoms in the protein was calculated using the first frame of the simulation as reference. From this frame, two Cα-atoms were chosen to determine the axis around which the twisting is measured. In HCMV, these were residue 135 of pUL50 and residue 113 of pUL53, both located in β-sheets. In the α-herpesviral NECs, the axis was defined between Cα of residues occupying the same positions in the structural alignment; in PRV residue 139 of pUL34 and residue 80 of pUL31, and in HSV-1, residue 153 of pUL34 and residue 113 of pUL31.

To avoid rotation or translation of the axis throughout the trajectory, an RMSD fit was performed on the subsequent frames. Each atom in each frame was assigned its own vector normal to the axis. For each vector, the angle between itself and the respective vector in the reference structure was calculated. In each frame, the moduli of all angles were summed up and divided by the number of vectors to obtain the average twist angle in each frame. Only backbone atoms were considered in this analysis.

### 3.4. Other Methods

Pairwise structure-based sequence alignments to identify homologous residues in the different viral NECs were obtained from the DALI server [42], and manually edited to obtain the multiple sequence alignment. For molecular visualization, the UCSF Chimera package was used [43]. Distance and root-mean-square deviation (RMSD) analyses were done using CPPtraj [44]. Occupancies of salt bridges and hydrogen bonds were calculated with VMD [45], with a distance cutoff of 3.5 Å. The distances were measured using CPPtraj, and simplified by only considering the shortest possible salt bridge distance between two sidechains. All scatterplots were coloured applying the same cutoff as above, and contain combined information of both MD runs.

## 4. Conclusions

Taken together, our study reveals that the isolated HSV-1 and HCMV NEC heterodimers differ significantly in the stability of the subdomain orientation, which results from structural differences in the secondary interfaces (Figure 3 and Figure 4). On the level of global Hexagon dynamics, HSV-1 still displays a slightly higher conformational stability resulting in a more regular Hexagon structure over the simulation time (Figure 9). Although nuclear budding can, in principle, be investigated by electron microscopy [46], there exist, to the best of our knowledge, no quantitative studies that compare the efficiency of the nuclear budding process between different herpesviruses. Since the differences in the dynamics are most pronounced at the level of the NEC heterodimers prior to Hexagon formation, one might speculate that they rather affect the earlier events of the nuclear egress, such as the interaction with NEC-associated proteins. For example, there are several nonhomologous auxiliary NEC-proteins [24] which might be recruited in a different fashion by α- and β-herpesviruses. In addition, even homologous NEC-associated kinases differ in their ability to phosphorylate cellular retinoblastoma and lamin A [47], which could reflect differences in recruitment by α- and β-herpesviral NECs.

However, we also detected common features in the structure and dynamics in the NEC assemblies of HCMV and HSV-1. In both systems, the oligomer interfaces observed between adjacent NEC heterodimers are remarkably weak, and are not significantly stabilized by the formation of a hexagonal ring-like structure (Table 1). In both viruses, the OIF I connecting adjacent pUL53 (pUL31) subdomains is particularly weak, which is in line with an enhanced mobility of these subdomains (Figure 8 and Figure 9). The latter common feature is particularly remarkable, because the respective dynamics might be of functional relevance either for the formation of curved structures or for the interaction with other proteins.

The formation of curved structures, instead of planar lattices, represents a requirement for nuclear vesicle formation during the budding process [48]. However, spherical coats displaying six-fold rotational symmetry can only be generated when hexagonal elements are interspaced by pentagonal symmetry elements or get disrupted in a different way. This principle does not only apply to the macroscopic patches of a soccer ball, but also to the icosahedral symmetry of herpesviral capsids [24]. However, no pentagonal NEC oligomers have been observed. Thus, the plasticity of the NEC Hexagons might facilitate the formation of curved coat structures from planar lattices in the absence of pentagonal elements.

Alternatively, the dynamics of the Hexagons might allow for an induced fit interaction with other proteins. This hypothesis appears particularly attractive for the recognition of the virus capsid, which displays hexagonal surface structures, termed major capsid protein (MCP) hexamers [49]. The spacing of the MCP hexamers is larger than that of the NEC Hexagons in the lattice, suggesting that rather isolated NEC Hexagons, and not the intact lattice, are involved in this interaction [24]. The interaction site between NEC and capsids was mapped to K242 within helix10 of PRV pUL31 [50]. In this context, the pronounced flexibility, detected for the pUL53 (pUL31) subdomain in our study, (Figure 9) might be relevant to allow for an induced fit between the hexagonal NEC and MCP units.

A further dissection of the putative functional roles of NEC Hexagon flexibility outlined above will require further experimental and computational studies. For a computational investigation of the interaction between NEC and capsid, docking studies and subsequent MD simulations might be applied using the available atomic structures of HCMV NEC [19,22] and capsid [49]. Investigation of larger assemblies from the lattice (e.g., of seven hexagonal rings; Figure 2) by MD simulations appears demanding, due to the large size of the systems, but should be feasible on high-performance computing systems. Such simulations should give insight into whether the lattices remain planar in solution, or a curvature occurs spontaneously. Alternatively, restraints may be used to induce curved structures and to study the mechanical properties of lattices.

In summary, our work has shown that Hexagons from HCMV and HSV-1 exhibit a common region of enhanced dynamics, and the resulting conformational plasticity offers an explanation for the formation of curved vesicle structures or the recognition of hexameric capsid proteins. Since the nuclear egress is a process that is unique to herpesviruses, it also presents a promising target for the design of drugs that selectively interfere with viral replication. The present study has revealed that the interfaces in higher NEC oligomers are, conformationally, rather labile. Thus, a disruption of these interactions by a drug appears feasible. Modulation of protein oligomerization by small molecules has become an emerging approach in drug design that is currently tested in numerous diseases [51] and might also be applied to the herpesviral NEC in the future.

## Figures and Tables

**Table 1:** Stability of the polar interactions in the oligomer interfaces. The analysis was performed for a DimerDimer (“DimDim”) and the Hexagons (“Hex”). Residues belonging to pUL53 (pUL31) are indicated in italics. All values give the percentage of the simulation time, for which the respective interaction was observed.

Interface	Inter-Action	HCMV		Inter-Action	HSV-1	
		DimDim	Hex		DimDim	Hex
OIF I	*E282R225*	16	14	*E302K262*	35	30
OIF II	*E115*R27	64	77	*S250*G91	67	46
OIF III	E156K36	53	58	R139S48	61	43

## Supplementary Materials

Supplementary materials can be found at http://www.mdpi.com/1422-0067/19/10/2908/s1.

## Abbreviations

MD	molecular dynamics
HSV-1	herpes simplex virus 1
PRV	pseudorabies virus
HCMV	human cytomegalovirus
INM	inner nuclear membrane
ONM	outer nuclear membrane
ER	endoplasmic reticulum
cryo-EM	cryo-electron microscopy
NEC	nuclear egress complex
PIF	primary interface
SIF	secondary interface
OIF	oligomer interface
MCP	major capsid protein
RMSD	root-mean-square deviation
RMSF	root-mean-square fluctuation
PDB	protein data bank
