# Supplementary material for: Conformational Dynamics of Herpesviral NEC Proteins in Different Oligomerization States
Source: Int J Mol Sci. 2018 Sep 25;19(10):2908. doi: 10.3390/ijms19102908 (PMC6213152; doi:10.3390/ijms19102908)
Supplement: Supplementary file 1 [file ijms-19-02908-s001.zip › NEC_supplement_230818.pdf]

Supplementary Materials

Conformational Dynamics of Herpesviral NEC Proteins in Different Oligomerization States

Benedikt Diewald [1], Eileen Socher [1], Christian A. Söldner [1] and Heinrich Sticht [1,*]

[1] Division of Bioinformatics, Institute of Biochemistry, Friedrich-Alexander-University Erlangen-Nürnberg (FAU), Fahrstraße 17, 91054 Erlangen, Germany, benedikt.diewald@fau.de, eileen.socher@fau.de, christian.soeldner@fau.de

* Correspondence: heinrich.sticht@fau.de

Received: date; Accepted: date; Published: date

Keywords: molecular dynamics, HCMV, HSV-1, PRV, nuclear egress complex, alpha-herpesvirus, beta-herpesvirus

---

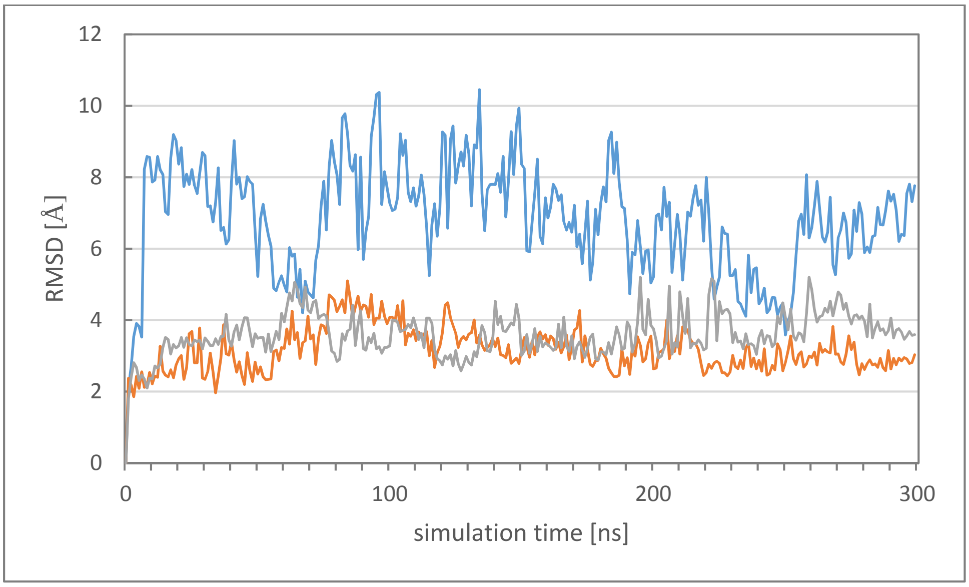

Figure S1. Plot of the RMSD of the HCMV NEC (blue), the HSV-1 NEC (orange), and the PRV NEC (grey), of run2 of the heterodimer simulations.

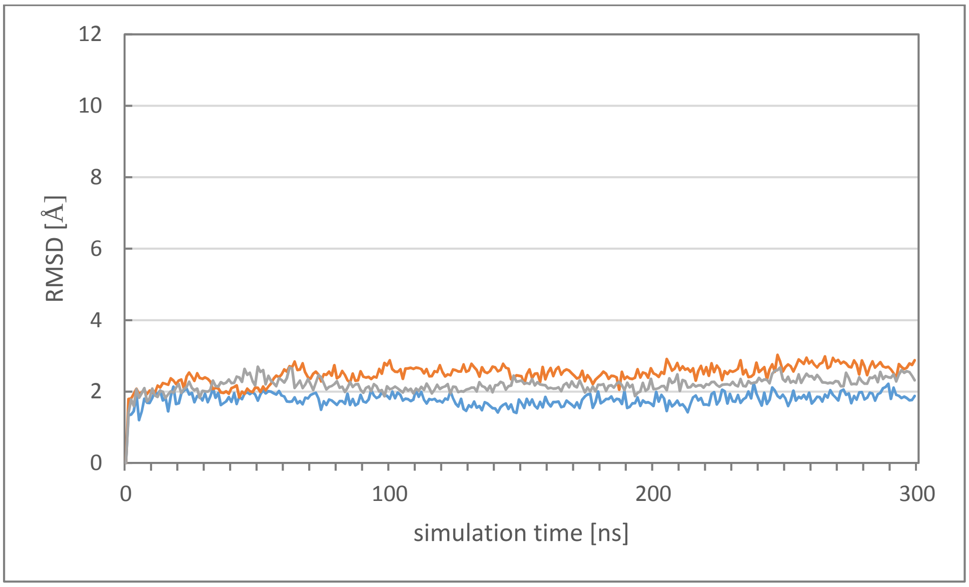

Figure S2. Plot of the RMSD of the pUL53 (pUL31) subdomains of HCMV (blue), HSV-1 (orange), and PRV (grey), of run2 of the heterodimer simulations.

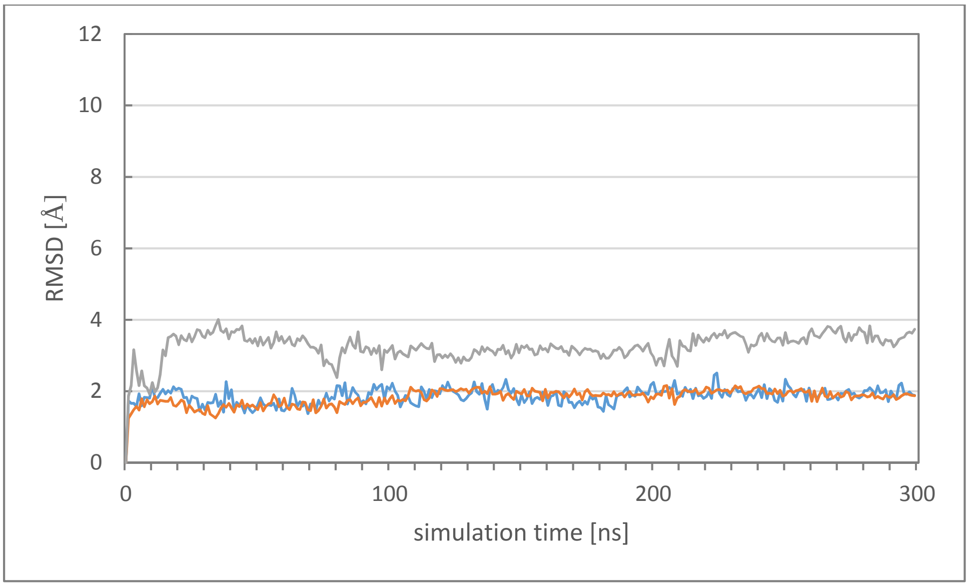

Figure S3. Plot of the RMSD of the pUL50 (pUL34) subdomains of HCMV (blue), HSV-1 (orange), and PRV (grey), of run2 of the heterodimer simulations.

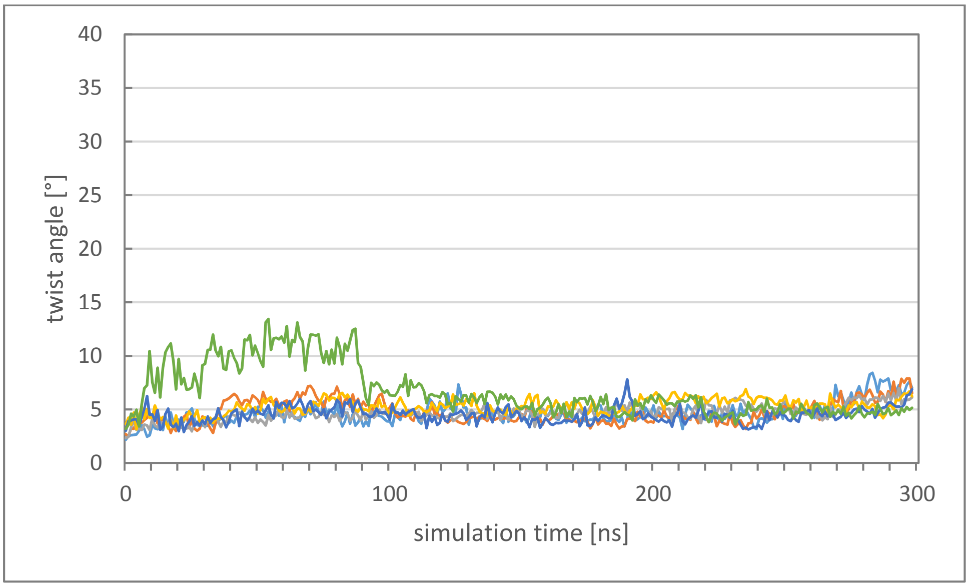

Figure S4. Twist angle of HCMV Hexagon simulation run2; the different colours mark the individual heterodimers.

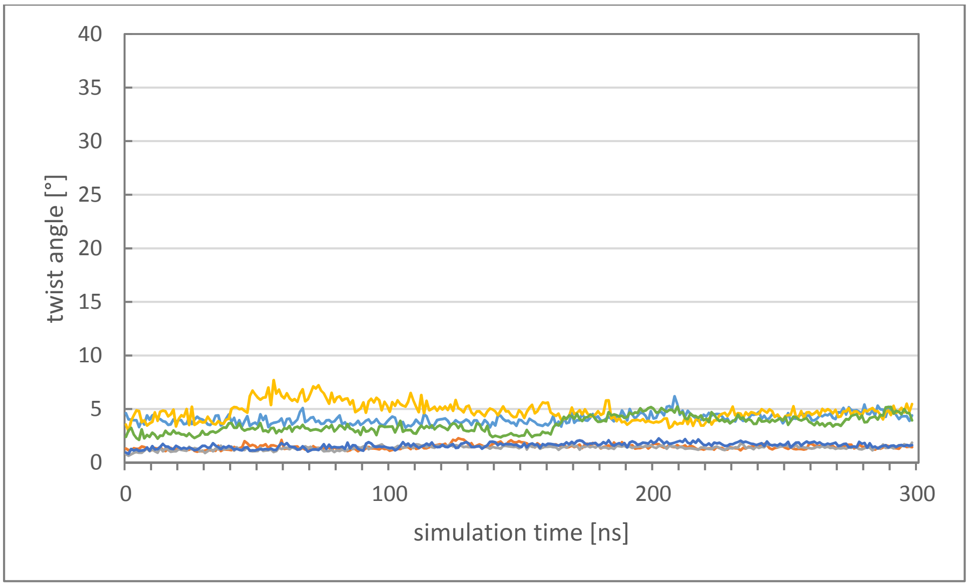

Figure S5. Twist angle of HSV-1 Hexagon simulation run2; the different colours mark the individual heterodimers.

Movie M1. The twisting of the HCMV NEC subdomains illustrated by a video containing simulation frames arranged in a fashion that displays a continuous twist. In the video the twisting is played forwards and backwards, so it can be watched in a loop.

Movie M2. This video shows the first 120 ns of run1 of the HCMV Hexagon simulation. All chains are coloured individually. Top-view on the pUL53 side with the pUL50 subdomains partially concealed.

Movie M3. This video shows the first 120 ns of run1 of the HSV-1 Hexagon simulation. All chains are coloured individually. Top-view on the pUL31 side with the pUL34 subdomains partially concealed.
